# Physiological mechanisms of the impact of heat during pregnancy and the clinical implications: review of the evidence from an expert group meeting

**DOI:** 10.1007/s00484-022-02301-6

**Published:** 2022-05-12

**Authors:** Louisa Samuels, Britt Nakstad, Nathalie Roos, Ana Bonell, Matthew Chersich, George Havenith, Stanley Luchters, Louise-Tina Day, Jane E. Hirst, Tanya Singh, Kirsty Elliott-Sale, Robyn Hetem, Cherie Part, Shobna Sawry, Jean Le Roux, Sari Kovats

**Affiliations:** 1 Department of Obstetrics and Gynaecology, Guy’s and St Thomas’ NHS Trust, London, UK; 2 Division of Paediatric and Adolescent Health, Institute for Clinical Medicine, University of Oslo, Oslo, Norway; 3 Department of Pediatrics and Adolescent Health, University of Botswana, Gaborone, Botswana; 4 Department of Medicine, Clinical Epidemiology Division, Karolinska Institutet, Stockholm, Sweden; 5 Medical Research Council Gambia at London School of Hygiene and Tropical Medicine, Fajara, The Gambia; 6 Centre On Climate Change and Planetary Health, London School of Hygiene and Tropical Medicine, London, UK; 7 Faculty of Health Sciences, Wits Reproductive Health and HIV Institute, University of the Witwatersrand, Hillbrow, Johannesburg, 2001 South Africa; 8 Environmental Ergonomics Research Centre, Loughborough Design School, Loughborough University, Loughborough, UK; 9 Department of Population Health, Aga Khan University, East Africa, Nairobi, Kenya; 10 Maternal, Adolescent, Reproductive & Child Health Centre, London School of Hygiene and Tropical Medicine, London, UK; 11 Nuffield Department of Women’s and Reproductive Health and the George Institute for Global Health, University of Oxford, Oxford, UK; 12 Climate Change Research Centre, University of New South Wales, Sydney, Australia; 13 Department of Sport Science, Sport, Health and Performance Enhancement (SHAPE) Research Centre, Nottingham Trent University, Nottingham, UK; 14 School of Animal, Plant and Environmental Sciences, Faculty of Science, University of the Witwatersrand, Johannesburg, South Africa

**Keywords:** Heat stress, Clinical medicine, Pregnancy, Labour, Pregnant women, Foetus, Newborn

## Abstract

Many populations experience high seasonal temperatures. Pregnant women are considered vulnerable to extreme heat because ambient heat exposure has been linked to pregnancy complications including preterm birth and low birthweight. The physiological mechanisms that underpin these associations are poorly understood. We reviewed the existing research evidence to clarify the mechanisms that lead to adverse pregnancy outcomes in order to inform public health actions. A multi-disciplinary expert group met to review the existing evidence base and formulate a consensus regarding the physiological mechanisms that mediate the effect of high ambient temperature on pregnancy. A literature search was conducted in advance of the meeting to identify existing hypotheses and develop a series of questions and themes for discussion. Numerous hypotheses have been generated based on animal models and limited observational studies. There is growing evidence that pregnant women are able to appropriately thermoregulate; however, when exposed to extreme heat, there are a number of processes that may occur which could harm the mother or fetus including a reduction in placental blood flow, dehydration, and an inflammatory response that may trigger preterm birth. There is a lack of substantial evidence regarding the processes that cause heat exposure to harm pregnant women. Research is urgently needed to identify what causes the adverse outcomes in pregnancy related to high ambient temperatures so that the impact of climate change on pregnant women can be mitigated.

## Background

Pregnant women and the fetus are increasingly recognised as being particularly vulnerable to the effects of extreme heat (Roos et al. 2021). There is mounting epidemiological evidence that high ambient temperatures are associated with pregnancy complications and adverse fetal and neonatal complications and outcomes including preterm birth, stillbirth, low birthweight (Zhang et al. 2017; Chersich et al. 2020), congenital anomalies (Haghighi et al. 2021), pre-eclampsia (Shashar et al. 2020), gestational diabetes (Pace et al. 2021) and emergency hospital admissions during pregnancy (Kim et al. 2019). There is also accumulating evidence of high temperatures negatively impacting the mental health of pregnant women, as well as altering their behaviour (Lin et al. 2017). Heat can be perceived as a barrier to engaging in outdoor activities (Caperchoine et al. 2009) and may deter women from physical activity. Additionally, many women have to work in high temperatures until late in their pregnancy (Spencer et al. 2022). However, there is a lack of evidence on the pathophysiological mechanisms and outcomes that are needed to inform clinical practice and public health strategies to manage heat risks and reduce impacts on mothers and babies.

Experimental animal studies, studies on cell lines and stem cells, are suitable for basic research of human physiology and the fetomaternal thermal relationship is thought to be similar across all mammals (Laburn 1996). Animal studies indicate that an elevation of maternal core temperature by 1.5 to 2 °C above the baseline, equivalent to 39 °C in humans, is a threshold above which there are teratogenic consequences for the fetus (Gericke et al. 1989; Ravanelli et al. 2018). These include neural tube defects, oro-facial anomalies and cardiovascular defects, among others (Graham 2020). The first trimester is considered a particularly vulnerable period for heat insult and the risk of congenital anomalies (Bell et al. 1986; Miller et al. 2002; Ravanelli et al. 2018). Due to the long gestation period in humans, and the complex interaction of different exposures throughout pregnancy, it can be difficult to discern the high risk exposures, including the timing of the exposure, that cause adverse birth outcomes (Chersich et al. 2020).

Several hypotheses have been proposed to describe the physiological mechanisms by which high ambient temperatures cause clinical complications for the mother and fetus, and these will be discussed later in the article; however, currently there is very limited definitive evidence (Roos et al. 2021). Ethical considerations prevent interventional studies that examine harmful effects during pregnancy and will continue to do so. Therefore, evidence must be extrapolated from animal models, in vitro experiments and a limited number of observational studies.

The Earth’s climate is changing; average global surface temperatures are continuing to increase at a rate of 0.2 °C ± 0.1 °C per decade, resulting in higher seasonal temperatures and more frequent and intense heat waves (Hoegh-Guldberg et al*.* 2019). Extreme heat has the greatest effects in low resource settings where access to cooling may be limited for the poorest households and working in high ambient temperatures can be difficult to avoid (King and Harrington 2018). Populations in temperate climate zones are being exposed to more atypical hot weather, and with housing and behaviours that are not well adapted to the heat. In order to protect pregnant women and the fetus from the damaging effects of heat exposure, it is crucial that we establish a better understanding of the physiological mechanisms involved.

## Objectives

This evidence statement, based on a literature search and the findings of an interdisciplinary expert workshop, aims to examine how exposure to high ambient temperature affects the pregnant woman and fetus. The evidence surrounding thermoregulation in pregnancy will be reviewed, the effect of environmental heat load during pregnancy, childbirth and delivery will be explored, and current hypotheses that explain how heat exposure may trigger adverse birth outcomes, including low birth weight and preterm birth, will be examined.

The aim is to build a consensus on how high ambient temperatures impact women and the fetus during pregnancy and childbirth, based on the current scientific evidence. Additionally, this paper aims to identify the gaps in current knowledge and practise, and highlight areas that would benefit from future work.

## Methods

An expert group was formed of experts in thermal physiology, animal physiology, exercise physiology, maternal physiology, maternal and environmental epidemiology, neonatology and obstetrics.

In advance of the expert meeting, an exploratory review of the literature took place. A search was performed in April 2021 using the synonyms ‘pregnancy’ OR ‘pregnant’ OR ‘gestation’ OR ‘labour’ OR ‘maternal’ AND ‘heat’ OR ‘temperature’ OR ‘climate change’ OR ‘heatwave’ OR ‘seasonal’ OR ‘heat stress’ AND terms related to the condition under study (e.g., ‘preterm birth’, ‘foetal distress’). PubMed, Cochrane and Google Scholar were searched. Only references in the English language were included. In addition, the reference lists of existing clinical guidelines and identified articles were manually searched. From these results, relevant information was extracted to formulate an initial schematic of hypotheses and evidence to be discussed at the expert meeting, which was circulated for review by the working group.

The expert meeting reviewed the evidence for the following questions: (1) Is thermoregulatory control impaired in pregnant women? (2) Does exposure to extreme temperatures reduce blood flow to the placenta? (3) How does exposure to extreme heat cause preterm birth? (4) Does a high ambient temperature affect normal childbirth? The expert meeting took place virtually on 14th of June 2021 to discuss the findings, and shortly after the meeting, a full consensus draft was written collaboratively by the working group. 

## Results

### Thermoregulation in pregnancy

Core body temperature in adults is maintained within narrow margins and is dependent on the balance between internal heat production, capacity for heat loss to the environment and environmental heat load (Kurz 2008). Pregnancy induces numerous physiological changes in women in addition to changes in body mass. Cardiovascular changes occur gradually throughout pregnancy so that by the third trimester, plasma volume and cardiac output increase by almost 50% (Hytten 1985). The increase in cardiac output is initially due to increased stroke volume but by the end of the second trimester, a raised heart rate is the main component of this increase (Hall et al. 2011). Placental blood flow reaches 600–700 ml/min by the end of pregnancy and is not autoregulated; it is dependent on cardiac output and varies directly with systemic maternal blood pressure (Wang and Zhao 2010).

Physiological changes of pregnancy include adaptations that affect thermoregulation (Bonell et al. 2020; Dervis et al. 2021). Numerous protective adaptive measures exist including a reduction in core temperature, lower sweating threshold, an increase in plasma volume and skin blood flow and an increase in thermal heat capacity due to a rising body mass. These enable pregnant women to maintain their core temperature within normal limits (Clapp 1991; Lindqvist et al. 2003; Bonell et al. 2020) despite the physiological changes of pregnancy which would otherwise act to impede a pregnant women’s ability to dissipate heat to the environment such as increased body mass and increased fat deposition, a change in the surface area-to-mass ratio of the woman and an increase in endogenous heat production as a result of the metabolic effort of the fetus and placenta (Abrams et al. 1970; Clapp 1991; Bonell et al. 2020). Theoretically, these protective mechanisms could be overwhelmed during exposure to extreme heat resulting in an increased risk of heat strain in pregnancy (Wells 2002; Bonell et al. 2020).

Fetal core temperature is maintained at approximately 0.5 °C above maternal core temperature (Randall et al. 1991) and is dependent on maternal temperature, placental blood flow and fetal metabolism (Lindqvist et al. 2003). The majority of fetal heat dissipation occurs across the placenta and a lesser amount through the amniotic fluid and uterine wall (Wells 2002). An increase in maternal core temperature will affect the fetal-maternal temperature gradient and influence the transfer of heat to the fetus (Walker et al. 1969).

Studies have shown that short-term exposure to heat through exercise or in a sauna or hot bath does not raise a pregnant woman’s temperature over the teratogenic threshold of an increase in 1.5 °C; Ravanelli et al. (2018) demonstrated in a review paper that pregnant women can use a hot bath of 40 °C or a dry sauna of 70 °C for 20 min and maintain their temperature within safe limits. Furthermore, Smallcombe et al. (2021) recently demonstrated no systematic alteration in thermoregulatory capacity among pregnant women in the second or third trimester performing moderate intensity exercise for up to 45 min in the heat (32 °C, relative humidity 45%) as compared to non-pregnant controls. However, whether there are adverse effects of prolonged exercise or physical labour in a hot environment is not yet known and the temperature thresholds at which adverse effects may occur are not well described. It can be assumed that the effects of heat are worse in hot environments with high relative humidity where increased absolute water vapour pressure restricts evaporative cooling; it remains to be determined whether pregnant women are more vulnerable to such conditions than other adult populations.

### High ambient temperature and intrapartum maternal fever

Childbirth is a physically strenuous process that normally causes a slight increase in core temperature as a result of endogenous heat production; approximately 0.2 °C over 10 h (Frölich et al. 2012). Intrapartum maternal fever is defined as a temperature over 38 °C during labour. It can result from infectious or noninfectious causes, and is associated with a number of poor foetal and neonatal outcomes, and an increased operative delivery rate (Burgess et al. 2017).

A hot delivery room has been suggested by several authors to contribute to maternal fever (Apantaku and Mulik 2007; Frölich et al. 2012; Burgess et al. 2017). However, evidence from observational studies is lacking. The working group judged that there is insufficient evidence to conclude that pregnant women may develop an intrapartum fever as a result of high ambient temperatures during delivery. Further studies are needed to investigate this hypothesis and develop guidance regarding optimum delivery room temperatures. It is important to ensure that the temperature of the delivery room is appropriate for not only the mother, but also the neonate, who is at risk of developing neonatal hypothermia; a major cause of neonatal morbidity and mortality in resource poor settings where maintaining specific delivery room temperatures may be difficult (Kumar et al. 2009). Although WHO recommend room temperatures between 25 and 28℃ for delivery (WHO 1997), there has been no formal evaluation of the evidence to support this. The indoor temperature range should reduce heat loss in the infant whilst remaining a comfortable temperature for the labouring woman.

### Heat exposure and reduced placental blood flow

Adults maintain normothermia during heat exposure or exercise by sweating and increasing blood flow to the skin. The resulting rise in skin temperature increases heat loss via convection and radiation and also enhances evaporative capacity of the skin wetted by sweat. Part of this blood flow is redirected from the visceral organs to the skin (Crandall et al. 2008). Under extreme heat stress, this results in competition for available cardiac output which may have adverse effects (González-Alonso et al. 2008); for example, non-pregnant athletes have been shown to risk kidney damage during high workloads in the heat as a result of low renal perfusion rates (Omassoli et al. 2019) and workers exposed regularly to heat have an increased incidence of acute or chronic kidney disease (Flouris et al. 2018). The placenta is an end-organ similarly reliant on cardiac output for perfusion, and it has been hypothesised that during extreme heat exposure, placental perfusion may become reduced to allow increased blood flow to the skin (Wells 2002; Bonell et al. 2020). A chronic reduction in uteroplacental blood flow can result in foetal growth restriction and low birth weight (Krishna and Bhalerao 2011).

Animal studies investigating the response of uterine blood flow to heat exposure are inconclusive; chronic and extreme heat exposure in sheep and cows has been demonstrated to result in reduced uterine blood flow by up to 30% with an associated reduction in placental weight (Reynolds et al. 1985; Dreiling et al. 1991). However, other studies in sheep have shown an increase in uterine blood flow during exogenous heat stress, likely as a result of vasodilation (Laburn 1996). Evidence for the effect in humans is limited and mixed. Uterine vascular resistance has been shown to increase in response to heat stress in a small number of hypertensive pregnancies but not in healthy pregnancies (Pirhonen et al. 1994) and moderate heat stress from a sauna has been shown not to affect umbilical artery blood flow (Vähä-Eskeli et al. 1991a, b) or cardiac output in pregnancy (K. K. Vähä-Eskeli et al. 1991a, b). Studies examining the effect of a short but intense period of exercise, rather than heat stress, have demonstrated both a decrease in uterine blood flow (Erkkola et al. 1992), no change in placental blood flow (Rauramo and Forss 1988) and an increase in uterine blood flow when exercising (Jeffreys et al. 2006). The effect of heat exposure for pregnant women and the foetus who may be undertaking regular physical activity in a hot climate remains unclear.

The working group considers that although theoretically plausible, more work is required to establish whether placental blood flow is reduced during heat stress in humans, and whether the effect varies as the demands for placental perfusion increase with gestation. Additionally, it would be important to understand whether placental blood flow changes as a function of heat stress severity in order to identify whether there is a dose-dependent relationship and whether critical environmental thresholds exist.

### High ambient temperatures and preterm birth

Preterm birth is one of the leading causes of neonatal and under-five mortality, and in addition to low birthweight can have adverse effects both on neonatal outcomes and outcomes later in life (Saigal and Doyle 2008). A recent meta-analysis demonstrated that with every 1 °C rise in ambient temperature, the risk of preterm birth increased (OR 1.05), an effect that was even greater during a heatwave (Chersich et al. 2020). However, there is no clear consensus as to the pathophysiological mechanism by which this occurs and a number of theories were identified during the literature search which likely coincide. Furthermore, high ambient temperature has been associated with a number of pregnancy complications such as gestational diabetes (Pace et al. 2021) and pre-eclampsia (Shashar et al. 2020), which are independently considered risk factors for preterm birth (Behrman 2007).

#### Oxytocin and prostaglandin release

Prostaglandins (hormone-like peptides with various physiological functions) and oxytocin (a neurotransmitter and hormone) are both known to be involved in the initiation of childbirth (Blanks and Thornton 2003; Olson 2003). Animal studies have shown that heat stress triggers an increase in oxytocin secretion (Dreiling et al. 1991) and prostaglandin F2α release (Wolfenson et al. 1993). Consequently, it has been hypothesised that any rise in oxytocin and prostaglandins resulting from heat stress in humans could initiate childbirth (Dadvand et al. 2011). To our knowledge, no research has yet been conducted among pregnant women examining whether heat stress induces release of prostaglandins and oxytocin.

#### Oxidative stress and release of inflammatory markers

Heat strain in animals and non-pregnant adults can cause oxidative stress and the release of endotoxins, cortisol, adrenaline, cytokines and other inflammatory markers (Dreiling et al. 1991; McMorris et al. 2006; Selkirk et al. 2008; Wang et al. 2015). It has been suggested that this inflammatory cascade could trigger preterm labour as a result of inflammation at the maternal-foetal interface (Peltier 2003; Schifano et al. 2013), or a subsequent increased foetal and placental prostaglandin release (Gronlund et al. 2020) although this has not yet been confirmed in heat stressed pregnant women.

#### Heat shock proteins

Whilst present in low levels in normal conditions, exposure to heat stress and other noxious stimuli upregulates production of numerous heat shock proteins in all animals, including humans. Their main function is to enhance protection and recovery of heat-stressed cells (Hromadnikova et al. 2015). Whilst an exhaustive exploration of the family of heat shock proteins is beyond the scope of this manuscript, there are some important considerations to note. Certain heat shock proteins have been linked to a number of pregnancy complications including fetal growth restriction and preeclampsia, though their role in these pathologies is not fully understood (Fukushima et al. 2005). Other heat shock proteins have been shown to be involved in the regulation of myometrial contractility (Lajinian et al. 1997; MacIntyre et al. 2008) and raised levels of Hsp70 have been detected in women with preterm birth (Fukushima et al. 2005). It has been hypothesised that Hsp70 may induce the release of proinflammatory cytokines which are proteins that cause inflammation. Such inflammation at the maternal-fetal interface in utero may initiate preterm birth (Peltier 2003; Dadvand et al. 2011).

#### Dehydration

Heat exposure resulting in dehydration has been proposed as another trigger for preterm birth (Lajinian et al. 1997; Schifano et al. 2013; Gronlund et al. 2020). In addition to hampering evaporative heat loss and thereby exacerbating heat stress, two potential mechanisms for dehydration as a trigger have been suggested; the first is that the reduction in vascular volume associated with dehydration causes a reduction in uterine blood flow which may destabilise placental decidual lysosomes (cell organelles that contain hormones) and trigger prostaglandin release resulting in preterm birth as previously described (Guinn et al. 1997).

The second relates to antidiuretic hormone (ADH), a hormone involved in the retention of water to prevent dehydration (Lajinian et al. 1997; Gronlund et al. 2020). Dehydration is known to trigger a release of ADH from the posterior pituitary gland (Thornton 2010). In certain situations, oxytocin, produced by the same gland, is released simultaneously. It has been hypothesised that this could trigger preterm labour as previously described (Theobald 1959; Guinn et al. 1997). However, research to date has not demonstrated any benefit of providing intravenous fluids, which would theoretically hydrate and halt the production of ADH, to women with threatened preterm birth (Stan et al. 2013).

#### Uterine contractility

Heat stress acting as a direct trigger of uterine contractions has been suggested as another contributor to preterm labour mechanisms (Dadvand et al. 2011). A study in pregnant baboons demonstrated an increase in uterine activity in response to heat stress (Morishima et al. 1975), and two studies in pregnant women have shown that heat exposure causes a slight increase in uterine activity; however, this was not sufficient to trigger regular uterine contractions or labour (Morishima et al. 1975; K. Vähä-Eskeli et al. 1991a, b).

The working group reviewed the evidence for these mechanisms which largely come from theories extrapolated from studies in animals or non-pregnant adults. It is possible that a number of mechanisms overlap to trigger preterm birth following heat exposure; however, the expert group judged that there is currently insufficient evidence to accurately determine the processes involved.

### Heat and hypercoagulability in pregnancy

Heat strain has been shown to lower central blood volume and activate coagulation pathways in non-pregnant adults; therefore, hypercoagulability is induced in response to elevated body temperature (Strother et al. 1986; Crandall et al. 2008; Meyer et al. 2013). Pregnancy is a hypercoagulable state that increases the risk of thrombosis and other complications with resulting morbidity, and in severe cases, maternal death (James 2009). It can thus be speculated that pregnant women could be at a higher risk of developing thromboembolic complications following exposure to extreme heat. Furthermore, sympathetic activity increases during heat strain leading to lower central blood volume that impacts on blood perfusion dynamics and pressure (Franke et al. 2003; McMorris et al. 2006). Catecholamines are expected to modulate the coagulation system which may add to complications in pregnancy and impact on the foetus’ development. Complications in the parturient include placental abruption which is the commonest cause of disseminated intravascular coagulation (DIC), whereas amniotic fluid embolism is a commonly fatal obstetric complication caused by introduction of amniotic fluid into the maternal circulation. Both these conditions may be linked to hypercoagulability and a proposed increased risk due to elevated body temperature in heatwaves and periods of extreme heat. These associations have not been investigated. In addition, infection and systemic inflammatory syndrome (SIRS) in the pregnant or delivering woman, in a hot environment, can reduce the central blood volume (Wade et al. 2011; Niven et al. 2012). Sparse information exists on the effects of elevated body temperature and reduced central blood volume and hypercoagulability isolated from severe infections (Levi and van der Poll 2010).

## Conclusions

Although there is increasing recognition that pregnant women may be more vulnerable to the effects of extreme heat, and exposure to high environmental heat has been shown to be associated with adverse pregnancy outcomes, there remains uncertainty regarding the key physiological mechanisms that cause adverse outcomes. Whilst numerous hypotheses exist, there is a paucity of supporting data. A better understanding of the physiological processes involved would aid in directing future research for prevention and targeted interventions, and furthermore assist in guiding the development of policy decisions.

Strenuous physical activity, whether for work or exercise, in high temperatures entails a risk of heat injury in humans. Exercise in pregnancy is recommended due to its numerous beneficial effects including modifying placental development to increase placental blood flow during the first and second trimester (Jackson et al. 1995; Rodríguez and González 2014). The effect of prolonged exercise and chronic heat exposure that may occur among pregnant women who work outdoors in hot countries is currently unknown and requires further study. Research evidence is so far consistent with current advice, such as that from the International Olympic Committee, that pregnant women can safely exercise for short periods of time in moderate temperatures (Bø et al. 2016; Smallcombe et al. 2021).

Important questions remain to be investigated including what is the effect of chronic heat exposure during pregnancy; does heat exposure at different gestational ages cause different effects; can women at risk of heat strain be identified early; is there a predominant physiological mechanism that can be targeted for an intervention; do high ambient temperatures act synergistically in combination with other environmental risk factors, such as air pollution, on pregnant women; does heat-stress exposure exert an effect on pregnancy outcomes in a dose-dependent manner; and are women with pre-existing medical conditions or established pregnancy complications affected differently by extreme heat. It is important to understand the mechanisms by which babies are affected for interventions to avoid poor outcomes. Addressing these research gaps would have relevance for the clinical management of pregnant women in the heat.

Guidelines from obstetric representative bodies largely advise pregnant women to avoid exercising in the heat or prolonged use of hot tubs and saunas (CDC 2017; ACOG 2015; NHS 2019); however, there is a lack of consistent information with regard to environmental heat exposure from these same organisations (Wells 2002; Graham 2020). Some professional bodies and governmental health departments offer generic advice to pregnant women regarding staying cool, using air conditioning and maintaining an appropriate fluid intake. However, there is a lack of specific advice as to the definition of a dangerously hot ambient temperature, whether there is a length of safe exposure time, or whether there are vulnerable time points during pregnancy when women should be particularly alert to the dangers of heat exposure.

Current advice for pregnant women with regard to heat exposure is sparse, inconsistent and not evidence based. It is important that pregnant women, healthcare professionals providing their care and policymakers are informed of the risks of exposure to high ambient temperature during pregnancy and provide clear, evidence-based advice as to how to protect themselves and their unborn babies.
